# Caregiver alignment with triage acuity levels and drivers for discrepancy between caregiver assessment and triage acuity levels: a cross-sectional questionnaire based study

**DOI:** 10.1186/s12913-024-12163-w

**Published:** 2025-01-17

**Authors:** Noelie Lengeler, Carl Alessandro Starvaggi, Manon Jaboyedoff, Sophie Affentranger, Kristina Keitel

**Affiliations:** 1 Division of Paediatric Emergency Medicine, Department of Paediatrics, Inselspital, Bern University Hospital, Bern, Switzerland; 2 Department of Orthopaedic Surgery, Spitalzentrum Oberwallis, Brig, Switzerland; 3 Graduate School for Health Sciences, University of Bern, Bern, Switzerland; 4 Paediatric Infectious Diseases and Vaccinology Unit, Service of Paediatrics, Department Mother-Woman-Child, Lausanne University Hospital and University of Lausanne, Lausanne, Switzerland; 5 Department of Paediatrics, Children’s National Hospital, Washington DC, USA; 6 Department of Clinical Research, University of Bern, Bern, Switzerland; 7 Machine Learning and Optimization Laboratory, EPFL, Lausanne, Switzerland

**Keywords:** Triage, Paediatric emergency medicine, Caregiver assessment, Sociodemographic factors

## Abstract

**Background:**

Caregiver concern is the main driver to paediatric emergency departments visits. Understanding caregiver worries is crucial to guide patients to the most appropriate healthcare setting. Previous research shows mixed findings on the accordance between caregiver assessment and professional triage.

**Methods:**

We assessed data from two questionnaire-based studies conducted over 27 months in two tertiary paediatric emergency departments in Switzerland to compare caregiver perception of their child’s medical acuity and standard nurse triage. Furthermore, we examined socioeconomic factors associated with caregiver perception.

**Results:**

Our study of 2,126 children seen in the two paediatric emergency departments showed that caregiver assessment aligned well with the acuity assigned by professional triage in 89% (1,901/2,126) of cases. In 142 cases (7%, 142/2,126), caregivers rating their child’s severity higher than nurse’s triage while in 83 cases (4%, 83/2,126), they rated it lower. In an univariable analysis, we found that family’s difficulties paying bills (20% vs. 12%) and low maternal education (19% vs. 10%) were associated with a higher percentage of caregiver rating their child’s severity higher than nurse’s triage. Fever as the main complaint was associated with caregiver rating their child’s severity lower than nurse’s triage.

**Conclusions:**

This questionnaire-based study shows that caregiver’s assessment of the severity of the child and nurse triage are concordant in most situations. Our study sheds light on the association between caregiver assessment and professional triage in two paediatric emergency departments in Switzerland, revealing some of the factors leading to discordance. These factors most probably reflect health illiteracy. It is important that healthcare professionals recognize and address factors influencing caregiver assessments to facilitate accurate decision-making and enhanced paediatric emergency care outcomes.

**Supplementary Information:**

The online version contains supplementary material available at 10.1186/s12913-024-12163-w.

## Background

Caregiver concern is one of the main factors driving visits to paediatric emergency departments (PEDs), and low-acuity visits constitute a significant proportion of PED visits. Visits for low-acuity reasons may happen because of a higher perception of severity of the condition of the child, or for other reasons [1–5]. The proportion of low-acuity visits is increasing, contributing to increased strain on emergency healthcare services [1, 5–8]. Low-acuity visits are generally considered medical and surgical problems, which do not require emergency care but could rather be treated by a primary health care professional. Healthcare provider and caregiver dissatisfaction [9] as well as suboptimal care [10] are associated with low-acuity visits. Suboptimal care, meaning the inadequate management of patients, can be caused in this context by a disruption of the continuity of care, which is crucial for chronic conditions [11] and leads to unnecessary diagnostic testing and treatments [12] as well as longer waiting periods. Better understanding what worries caregivers is crucial to improving health care in a PED. Furthermore, recognizing and using caregiver concern could improve the early detection of conditions with non-specific symptoms, such as sepsis, thereby enhancing patient outcomes [13, 14].

Several studies have evaluated the relationship between caregiver perceived level of acuity and actual health risks in children, with mixed findings [1, 9, 15–17]. Research from the United States and England found close agreement between caregiver’s and healthcare professional’s triage assessments [15, 18]. In contrast, other studies from the United States, Lithuania, and Switzerland highlighted a discrepancy in urgency perception, finding that caregivers both overestimated and underestimated their children’s health needs [1, 9, 19]. Explanations for these mixed findings are currently missing, yet highlight the complexity of the issue.

There are multiple determinants that have been identified as being associated with low-acuity visits, including demographic and socio-economic factors such as the age and education level of caregivers [20], health literacy [21], the age and cultural background of the child [5, 22, 23], financial precarity [22, 24, 25], single caregivers [26] and caregiver employment status [27]. Little is known about the demographic- and socio-economic factors directly linked to caregiver worry.

Paediatricians in Switzerland serve as the primary providers of health care for children. Almost 80% of all preschoolers see a primary care paediatrician regularly [28]. In all major cities, PEDs are available and operate 24/7. It is obligatory for all residents of Switzerland, including children, to have health insurance. Individuals pay the premiums, with subsidies available for those with lower incomes. In 2024 the mean premium for children was 112 CHF per month [29, 30]. Additionally, caregivers are required to share the cost of the healthcare services they use. This contribution includes a fixed deductible, which can range from 0 to 600 CHF and is selected by the patient, as well as a 10% retention fee on any additional healthcare expenses. The higher the deductible, the lower the monthly premiums. For children, the maximum retention fee is 350 CHF per year. The median monthly disposable income is 3,930 CHF [31].

This study aims to further explore the alignment between caregiver concern and triage acuity levels, as well as to identify socio-economic factors and health conditions correlating with a discrepancy between nurse triage and caregiver perception in two major Swiss PEDs.

## Methods

This study aimed to assess caregiver perception of their child’s medical acuity compared to standard nurse triage in the PEDs of two Swiss tertiary hospitals in Bern and Lausanne. Additionally, we examined demographic features and socioeconomic status as well as the main complaint “fever”, that might influence the accuracy of caregiver perception.

The University Hospital of Bern is located in the German-speaking region of the country, and the University Hospital of Lausanne is located in the French-speaking region. Both PEDs provide comprehensive care for medical and surgical conditions, staffed around the clock by paediatricians and paediatric emergency medicine specialists. They are the sole PEDs in their regions, collectively managing over 50,000 patient visits annually. The catchment area for the Bern PED is roughly 400,000 children, both urban and rural, with approximately 27,000 visits each year. Meanwhile, the Lausanne PED serves about 320,000 children and handles more than 30,000 visits annually [25].

This manuscript summarizesdata using a questionnaire that was previously conceived for two questionnaire-based prospective cohort studies. The “Infokids + ” study aimed to validate a paediatric acuity risk stratification algorithm in Bern. The PEDLAC (“Paediatric Emergency Department Low Acuity visits) study, sought to identify reasons leading to low-acuity PED visits in a cross-sectional, questionnaire-based study conducted in Bern and Lausanne [25]. Data from both studies were used to increase the sample size and to represent the two most dominant linguistic areas in Switzerland.

### Participants

This analysis includes children from the two questionnaire-based cohort studies with a recorded level of caregiver concern, socio-demographic status, and nursing triage level. Both studies included children visiting the PEDs, aged 0 to 16 years and only in Bern for the Infokids + and aged 0 to 18 years in Bern and Lausanne for the PEDLAC study. The only exclusion criteria was not being able to understand French, German, Italian, English, Albanese, Portuguese, Spanish or Tigrigna as the questionnaire was translated into these languages as the main languages spoken by caregivers in our PEDs. Upon initial contact with the PEDs, the patient’s caregivers were invited to participate in the studies when the research teams were present. During the time that the study team was not available, no participants were recruited. If agreeing to participate, the caregivers were then requested to fill in an informed consent. If a child was critically ill, patient care was placed above study inclusion. To ensure a representative patient sample, the study teams were available at various hours of the day, including weekend and night shifts.

**Fig. 1:**
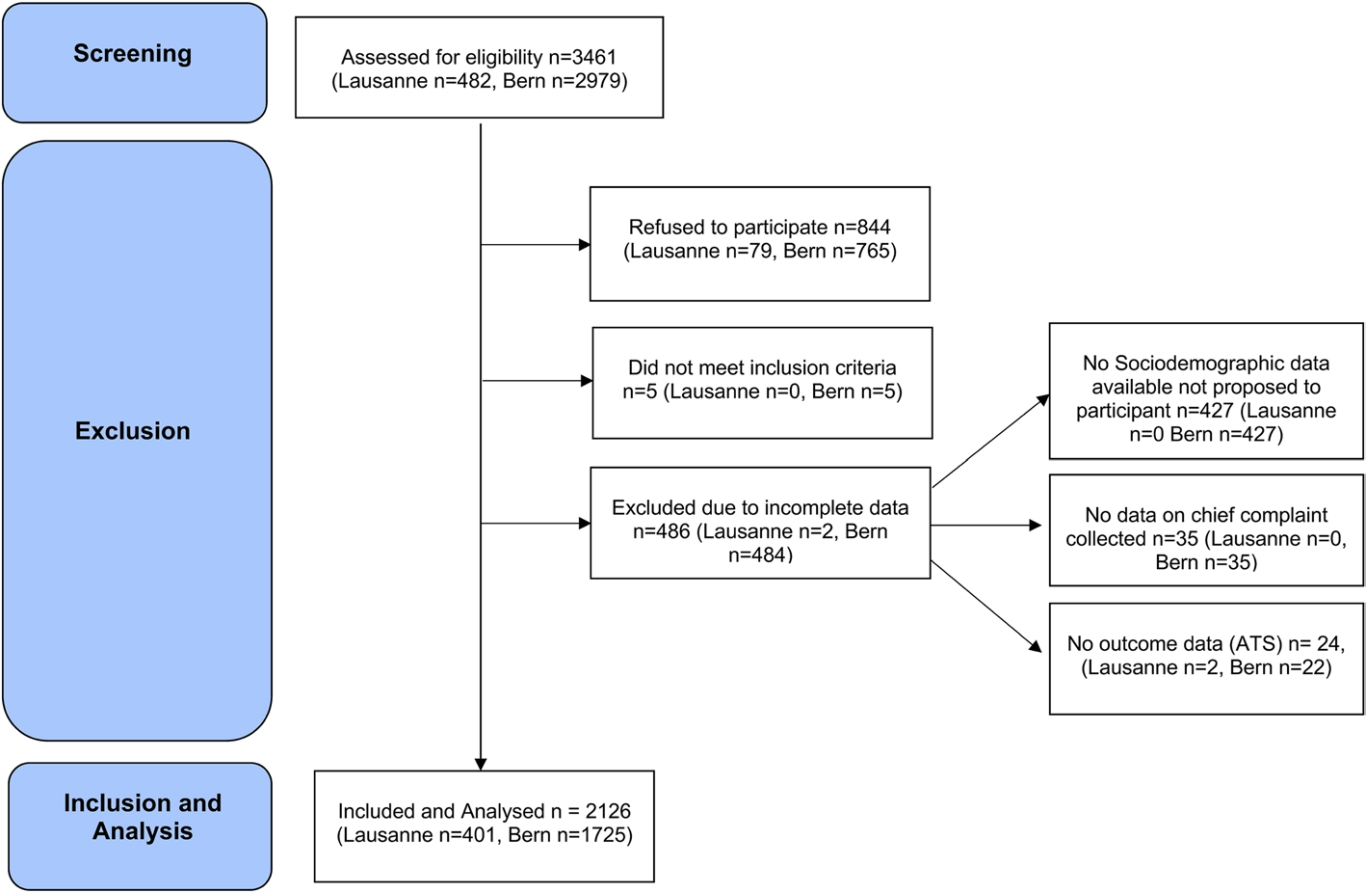
Flowchart of participants

The data collection for the PEDLAC took place between September 2019 and July 2020, whilst the data collection for the InfoKids + study took place from June 2020 to January 2022. The questionnaire including questions about demographic features and socioeconomic status was part of the PEDLAC study. During the overlapping data collection period between June and July 2020 all recruited participants filled in both questionnaires (8.0% (171/2126). After completion of the PEDLAC data collection, the socioeconomic questions were not initially collected. After a first analysis of the PEDLAC data showed an association between socioeconomic factors and low-acuity PED visits, the InfoKids + study team decided by late August 2020 to update the InfoKids + questionnaire to include key socioeconomic questions. We excluded participants with missing data needed for our analysis (Fig. 1). The 427 excluded participants were all recruited between July and August 2020, in the time span in which only the InfoKids + questionnaire without socioeconomic questions was used.

### Test methods

Caregivers were asked, in the PED, to retrospectively rate their perceived level of acuity at home, before deciding to come to the PED. In the PEDLAC study, we used a categorical scale, offering the options to rate the severity of the condition of the child as “mild”, “moderate” or “severe” [25]. In the InfoKids + study, we used the Likert scale from 0 (indicating no worry) to 10 (representing very high concern). We transformed data from the Likert scale into the categorical scale as follows: mild (0–3), moderate (4–8), severe (9–10) (Suppl. Table 1). We used the level of acuity assigned by the triage nurse upon patients’ arrival, based on the Australasian Triage Scale (ATS) as a reference index test. The ATS defines five levels of acuity: 1) requires immediate attention by a physician, 2) should be seen within 10 min, 3) requires attention within 30 min, 4) requires attention within 60 min, and 5) can wait up to 120 min for attention. The Australian Triage Score (ATS) is a widely used system in emergency departments to prioritize patient care based on the severity of their condition [32].

### Analysis

**Fig. 2:**
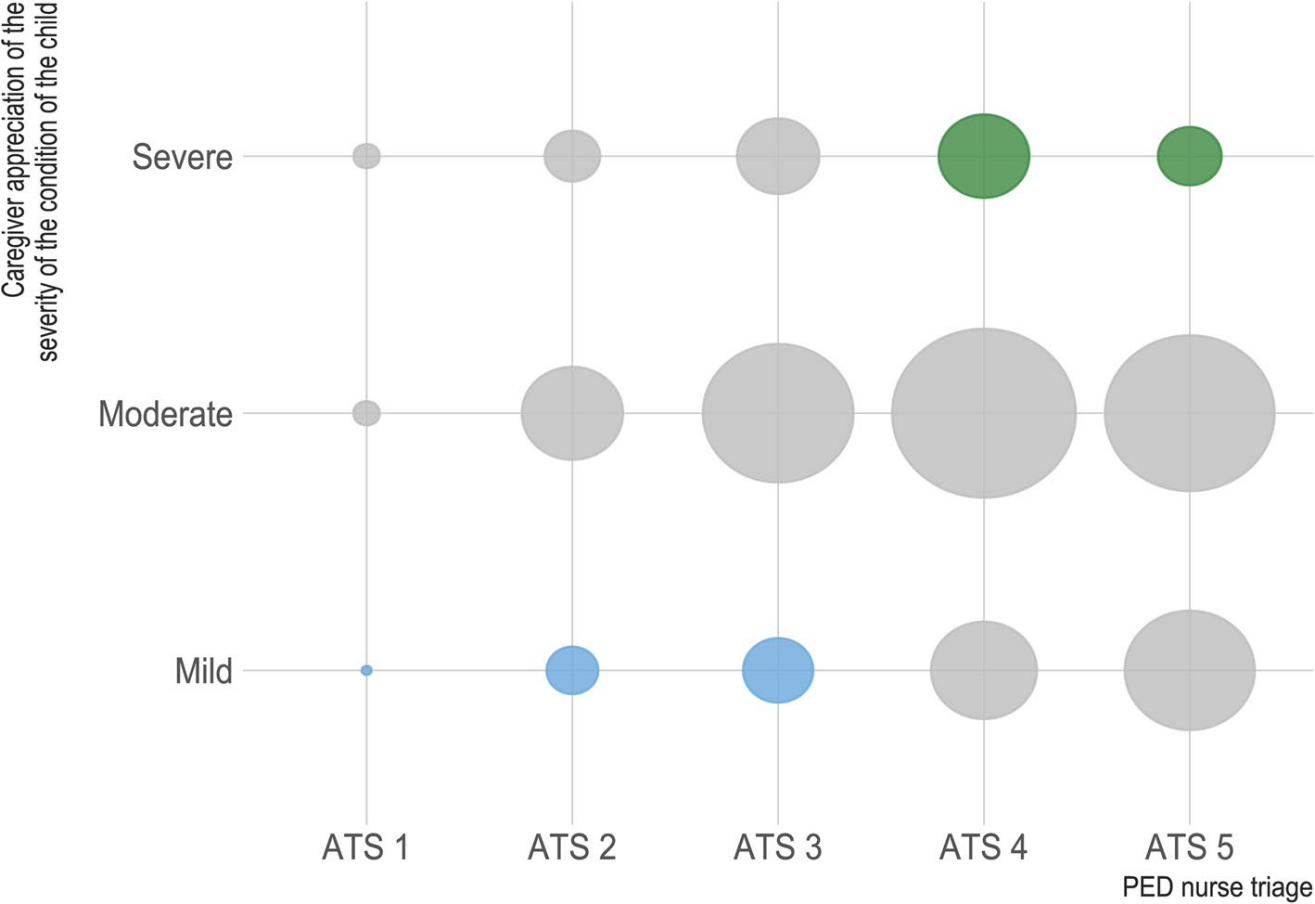
Caregiver appreciation of the severity of their child’s health in relationship with ATS value. Bubble size is proportional to the number of participants. Green bubbles represent over- and blue bubbles underestimation of the severity of the child by the caregiver

Data analysis was conducted using Stata®MP 16 (StataCorp, The College Station, Texas, USA). For continuous numeric variables mean (incl. 95%-CI), SD (incl. min. and max.), and median (incl. IQR) were calculated. For categorical variables, frequencies and percentages were calculated for the quantitative analysis. We compared characteristics of discrepant and concordant evaluation using Pearson’s Chi-square test. To identify factors associated with discordant evaluations, all statistically significant variables *p* < 0.05 in univariable analysis were included in an exploratory binary multiple logistic regression model(Suppl. Table 2). Statistical significance was set at *p* < 0.05. Given that a moderate perceived level of acuity could correspond to either high or low-acuity cases, our analysis focused on the mild and severe levels of acuity (Fig. 2). We defined caregiver overevaluation as a severe level of acuity perceived by caregivers and a low urgency triage reference score (ATS scores of 4 or 5). Conversely, low perceived level of acuity by caregivers and a high urgency ATS score of 1 to 3 was defined as caregiver underevaluation.

### Ethics

Both cohort studies were approved by the Cantonal Research Ethics Committees of Cantons of Vaud (Commission cantonale d’éthique de la recherche sur l’être humain CER-VD) and Bern (Kantonale Ethikkommission Bern) (project number 2019–00538 and 2019–02280). They were conducted in accordance with the ethical standards of both Ethics Committees and with the principles of the Declaration of Helsinki. Informed consent was obtained from the legal guardian at the time of PED visit for all participants.

## Results

The two cohort studies assessed 3,461 patients for eligibility, of which 844 (24.4%, 844/3,461) refused participation, 5 (0.1%, 5/3,461) did not meet inclusion criteria and 486 (14.0%, 486/3,461) were excluded due to incomplete data. The remaining 2,126 (61.5%, 2,126/3,461) patients were enrolled (Fig. 1). Participants were recruited between 5 pm and 8am in 26.4% (561/2,126) of cases, when primary health care services are unavailable. The median age of participants was 5 years (IQR 1–10), among them 15 (1%, 15/2,126) were infants younger than 1 month, 244 (12%, 244/2,126) were 1–11 months old, the remaining 1,867 children (87%, 1,867/2,126) were one year old or older with 0.6% (13/2,126) being 17–18 years old. Gender was equally distributed (48%, 1,017/2,119 female, 52%, 1,109/2,119 male). Caregivers indicated having difficulty paying bills in 12% (215/1,797) of cases. Maternal education was low (no schooling or only mandatory schooling) in 10% (214/2,084) of cases. Children were born in Switzerland in 97% (1,911/1,979), their mothers in 75% (1,239/1,652) and their fathers in 74% (1,221/1,643) of cases. Patients presented with a fever as their main concern in 13% of cases (267/1,947) (Table 1).

**Table 1:** Characteristics of the families over- and underestimating the severity of their child’s health condition

Characteristics	All participants		Missing data	Overestimation severity of child’s health condition by caregiver		Missing data	*p*-value^§^	Underestimation severity of child’s health condition by caregiver		Missing data	*p* value^§^
Total number	2126	(100%)	NA	142	(7%)	NA	-	83	(4%)	NA	-
Median age (years, IQR)	5	(1–10)	12 (1%)	4	(1–10)	1 (1%)	0.895^†^	5	(1–11)	1 (1%)	0.930^†^
Age: < 1 month	15	(1%)	NA	0	(0%)	-	0.310	**4**	**(5%)**	-	** < 0.001**
Age: 1 – 11 months	244	(12%)	NA	15	(11%)	-	0.804	13	(16%)	-	0.187
Age: 12 – 23 months	289	(14%)	NA	26	(18%)	-	0.103	9	(11%)	-	0.430
Age: 2 – 5 years	564	(27%)	NA	36	(25%)	-	0.758	17	(20%)	-	0.208
Age: 6 – 11 years	603	(29%)	NA	34	(24%)	-	0.226	20	(24%)	-	0.379
Age: 12 – 18 years	399	(19%)	NA	30	(21%)	-	0.488	19	(23%)	-	0.334
Gender: Female	1017	(48%)	7 (0%)	75	(53%)	1 (1%)	0.147	32	(39%)	1 (1%)	0.124
ATS 1	11	(1%)	0 (0%)	NA	-	-	-	1	(1%)	-	-
ATS 2	195	(9%)	0 (0%)	NA	-	-	-	27	(32%)	-	-
ATS 3	458	(22%)	0 (0%)	NA	-	-	-	55	(66%)	-	-
ATS 4	747	(35%)	0 (0%)	98	(70%)	-	-	NA	-	-	-
ATS 5	715	(34%)	0 (0%)	44	(30%)	-	-	NA	-	-	-
Difficulties paying bills	215	(12%)	329 (15%)	**24**	**(20%)**	19 (13%)	**0.007**	3	(4%)	15 (18%)	0.051
Maternal education: No school or mandatory only	214	(10%)	42 (2%)	**27**	**(19%)**	2 (1%)	** < 0.001**	5	(6%)	1 (1%)	0.188
Child born in Switzerland	1911	(97%)	147 (7%)	123	(95%)	13 (9%)	0.453	76	(96%)	4 (5%)	0.877
Mother born in Switzerland	1239	(75%)	474 (22%)	**55**	**(58%)**	47 (33%)	** < 0.001**	**63**	**(86%)**	**10 (12%)**	**0.019**
Father born in Switzerland	1221	(74%)	483 (23%)	**54**	**(60%)**	52 (37%)	**0.001**	61	(81%)	8 (10%)	0.154
Fever: yes	267	(13%)	179 (8%)	25	(17%)	0 (0%)	0.138	**5**	**(6%)**	0 (0%)	**0.041**

All proportions *p*-value for comparison of population overestimating the severity of the child with all participants (Pearson’s chi-square test if not otherwise specified)

*NA* non-applicable

^§^*p*-value for comparison of population underestimating the severity of the child with all participants (Pearson’s chi-square test if not otherwise specified)

^†^Student’s t-test

Figure 2 visualizes the caregiver perceived severity of their child’s health condition in relationship with the ATS score their child received at triage in the PEDs. The majority of caregivers (59%, 1,253/2,126) rated the severity of their child’s condition as “moderate”, whilst 23% (436/2,126) rated their child’s condition as “mild” and 11% (258/2,126) as “severe” (Suppl. Table 3). A minority of caregivers (8%, 179/2,126) did not give a perception of acuity by choosing “I do not know, or I do not want to answer”. In 89% (1,901/2,126) of cases the caregivers’ assessments aligned with the ATS scores assigned by the triage nurse. Instances of caregiver overestimation were identified in 7% of cases (142/2,126), when the child’s condition was perceived as “severe” but triaged as low-acuity (ATS 4–5). Conversely, caregiver underestimation was observed in 4% of cases (83/2,126), where the condition was judged “mild” by caregivers but triaged as urgent (ATS 1–3).

Several demographic and socio-economic factors were linked to both overestimation and underestimation of children’s health conditions by caregivers. Caregiver factors associated with overestimation of the severity of the child were caregiver foreign country of birth, low maternal education and having difficulties paying bills.

The child’s age (median age 4 vs. 5 with the same IQR [1–10]) and gender (53% vs. 48%), the child being born in Switzerland (95% vs. 97%), and fever (17% vs. 13%) as the primary complaint was not associated with caregiver overestimation.

Caregiver factors associated with underestimation of the severity of the child were families where the mother was born in Switzerland. Caregiver underestimation was significantly higher in children younger than 1 month. Caregivers of children presenting to the emergency room with a fever as their main complaint, were less likely to underestimate their child’s health condition.

Factors such as financial precarity (4% vs. 12%), low maternal education (6% vs. 10%), Switzerland being the birthplace of the child (96% vs. 97%) or the father (81% vs. 74%) were not significantly associated with caregiver underestimation.

We performed an exploratory multivariate analyse to evaluate potential co-dependence of characteristics associated with caregiver over- and underestimation and to determine which were the most significant factors. We found that caregiver overestimation was significantly reduced when the mother was born in Switzerland (OR = 0.35, 95%CI 0.15–0.84). Caregiver underestimation was significantly increased with children under 5 years of age (OR = 2.24, 95%CI 1.09–4.60) (Suppl. Table 2). However, complete data were only available for 33% (694/2,126) of patients, leading to the decision to not include these findings in our main results.

## Discussion

### Main findings

Our study showed that in the majority of instances, caregiver assessment did not diverge from the severity assigned at triage. Nonetheless, there remains 11% of all cases in which caregivers either underestimated (4%) or overestimated (7%) the acuity of their child’s condition. Demographic and socio-economic factors that were found to be associated with overestimation of severity were financial precarity, low maternal education and caregivers born abroad. Factors associated with underestimation of severity were children younger than 1 month and the mother being born in Switzerland. Fever as a chief complaint was associated with less caregiver underestimation.

### Strengths and limitations

The main strength of this study is that we were able to prospectively collect a large data sample from two tertiary-care PEDs in Bern and Lausanne. With two different locations and linguistic areas, they cover approximately 40% of the Swiss PED catchment area [5]. We were able to use the ATS Score as an index test, which, as with all triage systems, is an approximation and does not perfectly reflect the true acuity of a health condition but still allows a robust comparison. A Meta-Analysis found that the ATS, with a pooled reliability coefficient of 0.428, exhibited an acceptable level of overall reliability in emergency departments [33]. For severe Sepsis, it showed a sensitivity of 71% [34]. However, Ebrahimi et al*.* showed the reliability of the ATS may not be as effective for children as it is for adults [33].

Our study offers insight into the association between caregiver assessment and triage in a PED, as well as the association between demographic and socioeconomic variables associated with caregiver over- and underestimation of their child’s health condition. To our knowledge, this hasn’t been studied in Switzerland previously. Furthermore, we were able to link these factors to caregiver underestimation, which has not previously been studied in a PED.

There were several limiting factors in our study. Firstly, our measurement of caregiver assessment was limited to caregivers who were already in the emergency room. This may have introduced a selection bias, therefore potentially reducing the generalizability of our findings to the general public. Furthermore, a certain recollection bias could have occurred as the caregivers were asked in the PED how worried they had been about their child’s health condition at home. It is possible, that caregivers were on one hand reassured after having had their child assessed by health care professionals or on the other hand, that they were even more worried due to the overall experience in the emergency room. Our definition of underestimation should be used with caution, as caregivers who understated their child’s health condition by our definition had still recognized their child needed urgent medical care and had sought out the PED.

Secondly, some of the socio-economic variables had a high percentage of missing data largely because these questions were introduced later into the questionnaire in Bern. This may have introduced a bias in our study. However, since we did not change the recruitment strategy in Bern before and after introduction of the additional questions, a systemic bias is unlikely. The highest percentage of missing data was with paternal education, resulting in our decision to not report on it in our results.

Thirdly, it is important to note that the study was conducted before, during and after the COVID-19 pandemic. Based on internal data from our PEDs, the pandemicinitially led to a decrease in visits in 2020, mainly due to a decrease in high-acuity cases. In contrast, after the suspension of most COVID-19 measures the following year, visits increased, mainly due to a rise in low-acuity cases [35].

Fourthly, this study was conducted in tertiary-care hospitals, and therefore, its results may not be generalizable to regional non-academic PEDs, which have limited access to subspecialties and often refer more complex cases to tertiary care centres.

Lastly, it is important to note that there is a systematic error regarding the association of young age and caregiver assessment because neonates, by definition, receive an ATS level of 3 or less, meaning that all neonate visits were classified as urgent.

### Comparison to previous research

Previous studies comparing the perceived level of acuity by caregivers on presenting to a PED, with the level of acuity assigned at triage, were inconclusive. Kestner et al*.* assessed caregiver concern with the triage score given to their child at reception by a healthcare professional and found a close agreement of 44% [15], which is substantially lower than in our study. Huang et al*.* assessed how caregivers rated the urgency of standardized paediatric scenarios and found an overall good agreement between the participants and the physicians, although low-acuity scenarios were overestimated [36]. MacFaul et al*.* found caregiver assessment of illness severity correlated well with that of doctors [18]. In contrast, Burokiene et al*.* and Long et al*.* concluded that caregivers frequently perceived their child’s health condition to be more urgent than medical staff [1, 19]. Löflath et al*.* came to the same conclusion, adding that a significant proportion of children assessed as “low-acuity” by their caregivers did, in fact, require urgent care, showing misjudgement in both directions [9]. Similar to our study, Pehlivanturk-Kizlikan et al*.* compared caregiver assessment of their child’s emergency status using their triage tool, the PCTAS, yet measured a slight to no agreement between caregiver assessment and triage score [16].

Our findings align with some of the above-mentioned studies, but not all. A key reason behind this observed heterogeneity was that caregiver and healthcare agreement on the severity of a child’s health condition was defined differently in each study, with different methods and cut-offs, making comparison difficult.

Caregiver anxiety [3] and the feeling that their child needs urgent medical care have been found to be main reasons for low-acuity visits [2, 3, 37, 38]. However, we have to keep in mind that other factors, such as convenience and proximity to the PED [5, 39] and the belief that better care is provided in the PED [20, 40], also lead to low-acuity visits. Our study linked demographic and socio-economic factors to caregiver overestimation. This aligned with factors associated with low-acuity visits for financial precarity [22, 24, 25] and low maternal education [20]. We found that caregivers born outside of Switzerland were more likely to overestimate their child’s health condition. This may be due to cultural differences in health concepts.

Pehlivanturk-Kizilkan et al*.* measured caregiver overestimation directly, finding that children with maternal unemployment and paternal low level of education increased the likelihood of caregivers overestimating the emergency severity [16]. A comparison is not possible as we did not collect any or enough data on maternal unemployment and paternal low level of education.

In Switzerland, visits to a PED are typically more expensive than scheduled visits to a primary care provider. Qualitative interviews conducted by our group exploring this relationship in Bern found that the main reasons for families living with financial precarity and visiting the PED for low-acuity case were convenience factors due work schedules, wanting to avoid double costs occurring in case of a hospital referral, feeling that the PED was better value for money [41].

Fever is one of the most common complaints for low-acuity visits in PEDs, with multiple studies showing that caregivers worry about the harmful effects of fever [42, 43]. Fever has been shown to increase the likelihood of caregivers overestimating the severity of their child’s health [16]. However, we did not find an association between caregiver overestimation and fever, yet when children had a fever, caregivers were less likely to underestimate their health condition.

Our data showed that caregivers born abroad, as well as caregivers with lower education, were more likely to overestimate the severity of their child’s condition, which could indicate lower access to health literacy in these populations. A Swiss study found that 49% of the population regularly struggles to understand health information, with individuals facing financial precarity and those with lower educational levels being especially affected by this health literacy gap. Low-acuity visits and more frequent use of the health system have previously been associated with low health literacy [2, 44]. Better recognition of this provider-patient communication gap could potentially improve care for these vulnerable populations.

## Conclusion

In conclusion, our study sheds light on the association between caregiver assessment and professional triage in PEDs, showing accordance in most cases. Demographic and socio-economic factors associated with discordance between caregivers and healthcare professionals assessment were notably financial precarity, caregiver place of birth, and maternal education. Better recognition of this provider-patient communication gap could potentially improve care for these vulnerable populations and inform targeted public health interventions.

## Supplementary Information

Supplementary Material 1.

Supplementary Material 2.

Supplementary Material 3.

Supplementary Material 4.

## Data Availability

The datasets presented in this article are not readily available because they contain patient sensitive data. Requests to access the datasets should be directed to the corresponding author.

## Abbreviations

ATS: Australasian Triage Scale
PED: Paediatric Emergency Department
