# Supplementary material for: Caregiver alignment with triage acuity levels and drivers for discrepancy between caregiver assessment and triage acuity levels: a cross-sectional questionnaire based study
Source: BMC Health Serv Res. 2025 Jan 17;25:96. doi: 10.1186/s12913-024-12163-w (PMC11740441; doi:10.1186/s12913-024-12163-w)
Supplement: Supplementary file 1 — Supplementary Material 1. [file 12913_2024_12163_MOESM1_ESM.docx]

| **Parental**  **assessment** | **Parental level of worry** | | | | | | | | | | | |
| --- | --- | --- | --- | --- | --- | --- | --- | --- | --- | --- | --- | --- |
|  | **0** | **1** | **2** | **3** | **4** | **5** | **6** | **7** | **8** | **9** | **10** | **Total** |
| **Mild** | 5 (3%) | 10 (6%) | 7 (4%) | 7 (4%) | 4 (2%) | 3 (2%) | 2 (1%) | 1 (1%) | 0 (0%) | 0 (0%) | 0 (0%) | 39 (23%) |
| **Moderate** | 0 (0%) | 11 (6%) | 8 (5%) | 17 (10%) | 10 (6%) | 10 (6%) | 13 (8%) | 21 (12%) | 8 (5%) | 1 (1%) | 1 (1%) | 100 (59%) |
| **Severe** | 1 (0%) | 0 (0%) | 1 (1%) | 1 (1%) | 0 (0%) | 0 (0%) | 3 (2%) | 1 (1%) | 3 (2%) | 1 (1%) | 3 (2%) | 14 (8%) |
| **IDN*** | 1 (1%) | 1 (1%) | 2 (1%) | 0 (0%) | 1 (0%) | 5 (3%) | 2 (1%) | 3 (2%) | 2 (1%) | 1 (1%) | 0 (0%) | 18 (11%) |
| **Total** | 7 (4%) | 22 (13%) | 18 (11%) | 25 (15%) | 15 (9%) | 18 (11%) | 20 (12%) | 26 (15%) | 13 (7%) | 3 (2%) | 4 (2%) | 171 (100%) |
