# Supplementary material for: Caregiver alignment with triage acuity levels and drivers for discrepancy between caregiver assessment and triage acuity levels: a cross-sectional questionnaire based study
Source: BMC Health Serv Res. 2025 Jan 17;25:96. doi: 10.1186/s12913-024-12163-w (PMC11740441; doi:10.1186/s12913-024-12163-w)
Supplement: Supplementary file 2 — Supplementary Material 2. [file 12913_2024_12163_MOESM2_ESM.docx]

|  | **Overestimation** | | | **Underestimation** | | |
| --- | --- | --- | --- | --- | --- | --- |
| **Characteristics** | **Odds ratio** | **95% confidence interval** | | **Odds ratio** | **95% confidence interval** | |
| **Age <= 5 years** | 1.21 | 0.63 | 2.31 | **2.24** | **1.09** | **4.60** |
| **Gender** | 1.51 | 0.80 | 2.85 | 0.65 | 0.31 | 1.35 |
| **Difficulties paying bills** | 1.28 | 0.54 | 3.06 | 0.21 | 0.03 | 1.55 |
| **Maternal education: No school or mandatory only** | 1.26 | 0.49 | 3.23 | 0.99 | 0.21 | 4.70 |
| **Child born in Switzerland** | 0.64 | 0.16 | 2.54 | 0.30 | 0.03 | 3.07 |
| **Mother born in Switzerland** | **0.35** | **0.15** | **0.84** | 3.04 | 0.68 | 13.56 |
| **Father born in Switzerland** | 0.81 | 0.33 | 1.95 | 1.07 | 0.33 | 3.46 |
