# Supplementary material for: Caregiver alignment with triage acuity levels and drivers for discrepancy between caregiver assessment and triage acuity levels: a cross-sectional questionnaire based study
Source: BMC Health Serv Res. 2025 Jan 17;25:96. doi: 10.1186/s12913-024-12163-w (PMC11740441; doi:10.1186/s12913-024-12163-w)
Supplement: Supplementary file 3 — Supplementary Material 3. [file 12913_2024_12163_MOESM3_ESM.docx]

| **Parental Assessment** | **Australasian Triage Category** | | | | | |
| --- | --- | --- | --- | --- | --- | --- |
|  | **Category 1** | **Category 2** | **Category 3** | **Category 4** | **Category 5** | **Total** |
| **Severe** | 5 (0%) | 32 (2%) | 79 (4%) | 98 (5%) | 44 (2%) | 258 (12%) |
| **Moderate** | 5 (0%) | 125 (6%) | 295 (14%) | 449 (21%) | 379 (18%) | 1253 (59%) |
| **Mild** | 1 (0%) | 27 (1%) | 55 (3%) | 138 (7%) | 215 (10%) | 436 (21%) |
| **IDN*** | 0 (0%) | 11 (1%) | 29 (1%) | 62 (3%) | 77 (4%) | 179 (8%) |
| **Total** | 11 (1%) | 195 (9%) | 458 (22%) | 747 (35%) | 715 (34%) | 2126 (100%) |
