# Supplementary material for: Caregiver alignment with triage acuity levels and drivers for discrepancy between caregiver assessment and triage acuity levels: a cross-sectional questionnaire based study
Source: BMC Health Serv Res. 2025 Jan 17;25:96. doi: 10.1186/s12913-024-12163-w (PMC11740441; doi:10.1186/s12913-024-12163-w)
Supplement: Supplementary file 4 — Supplementary Material 4. [file 12913_2024_12163_MOESM4_ESM.docx]

| **Main Concern** | **All Participants** |
| --- | --- |
| **Trauma** | 717 (33.7%) |
| **Other (difficult to classify)** | 284 (13.4%) |
| **Fever** | 267 (12.6%) |
| **Gastrointestinal** | 164 (7.7%) |
| **ORL und dental** | 154 (7.2%) |
| **Respiratory Disease** | 115 (5.4%) |
| **Neurologic disease** | 102 (4.8%) |
| **Fluid and electrolyte disorders** | 77 (3.6%) |
| **Dermatological disease** | 74 (3.5%) |
| **Urinary tract disease** | 51 (2.4%) |
| **Musculoskeletal and connective tissue disease** | 41 (1.9%) |
| **Unknown** | 29 (1.4%) |
| **Ophthalmological disease** | 18 (0.9%) |
| **Mental health** | 10 (0.5%) |
| **Genital disease** | 9 (0.4%) |
| **Toxicological emergency** | 5 (0.2%) |
| **Haematological disease** | 3 (0.1%) |
| **Child abuse** | 2 (0.1%) |
| **Cardiovascular disease** | 2 (0.1%) |
| **Neoplastic disease** | 2 (0.1%) |
| **Total** | 2126 (100%) |
